# Separate and Combined Effects of Moderate-Intensity Exercise Training and Detraining with Protocatechuic Acid (PCA) on Myokines and Insulin-Signaling Pathways in Male Wistar Rats: A Preclinical Randomized Study

**DOI:** 10.3390/metabo15020087

**Published:** 2025-02-01

**Authors:** Hira Shakoor, Jaleel Kizhakkayil, Yauhen Statsenko, Carine Platat

**Affiliations:** 1 Department of Nutrition and Health, College of Medicine and Health Sciences, United Arab Emirates University, Al Ain 15551, United Arab Emirates; 201890012@uaeu.ac.ae (H.S.);; 2 Department of Nutrition and Exercise Physiology, Elson S Floyd College of Medicine, Washington State University, Spokane, WA 99202, USA; 3 Department of Radiology, College of Medicine and Health Sciences, United Arab Emirates University, Al Ain 15551, United Arab Emirates; e.a.statsenko@uaeu.ac.ae

**Keywords:** moderate-intensity training, detraining, myokines, protocatechuic acid, gastrocnemius muscle, glucose metabolism

## Abstract

**Background**: Exercise training positively modulates myokine secretion and improves glucose metabolism. Herein, we analyzed the effect of moderate-intensity training, detraining, and Protocatechuic Acid (PCA) supplementation on myokine secretions and regulation of insulin-signaling pathways. **Methods:** A five-arm study was conducted on 47 healthy male Wistar rats, trained at a moderate intensity level for four weeks (T0-T4). Animals were randomly classified into groups according to PCA supplementation and exercise durations: four weeks of Aerobic Training with or without PCA (AT4, AT4-PCA), eight weeks of Aerobic Training with or without PCA (AT8, AT8-PCA), and PCA Vehicle Control (VC). The animals were followed up until week 12 (T12). We decapitated six rats at T0 and T4, four rats per group at T8, and three rats per group at T12. Myokines (IGF-1, IL-6, FGF-21, myostatin, and irisin) were analyzed with ELISA. Western blot analysis measured protein expression of insulin-signaling pathways and GLUT-4 in the gastrocnemius muscle. **Results:** The IL-6 levels increased significantly (*p* < 0.01) with 8-week training in AT8 by 34% and AT8-PCA by 32%, compared to groups trained for only 4 weeks (AT4 and AT4-PCA). Similarly, the PI3K, and GLUT-4 expression improved in AT8 and AT8-PCA at T8. Training for 4 weeks improved IGF-1 levels, but a further 14% improvement was observed with 8-week training in AT8 at T8. Myostatin level significantly dropped by 27% even with 4-week training (*p* < 0.001). However, detraining increased the myostatin levels in all groups, but in AT8-PCA with PCA dose, myostatin reduced by 11% compared to AT8 at T12. PCA supplementation reduced the FGF-21 levels by 54% during detraining at T12 in AT8-PCA compared to AT8. However, the irisin level did not change markedly in any group. **Conclusions**: Physical training (with and without PCA) modulates myokine production and improves glucose metabolism, but the benefits are lost after detraining.

## 1. Introduction

Physical activity is a bodily movement produced by skeletal muscles that demands energy expenditure. At least 150 min of moderate-intensity aerobic physical activity every week is recommended for adults [1]. It is well documented that regular physical activity and endurance training are pivotal in improving glucose metabolism and insulin sensitivity, thus reducing the risk of type 2 diabetes (T2D) [2,3]. In this regard, the skeletal muscle plays an important role as it is one of the most metabolically active organs, comprising 40% of the total body weight [4], and is a primary site for glucose uptake and metabolism. Skeletal muscles are widely recognized for their critical role in maintaining metabolic health by improving muscle regeneration and maintaining blood glucose and lipid levels [5].

Exercise-induced muscle contractions trigger several key metabolic adaptations. These include enhanced glucose uptake and translocation of glucose transporter-4 (GLUT-4) and the activation of signaling pathways such as insulin receptor substrate (IRS) 1 (IRS-1), IRS-2, and associated phosphatidylinositol (PI) 3-kinase (PI3K), protein kinase B (Akt), and AMP-activated protein kinase (AMPK) [4,6]. Consequently, regular physical training regulates blood glucose levels and improves overall metabolic health. Furthermore, in our previous study, we observed a significant improvement in area under the curve (AUC) of the intraperitoneal glucose tolerance test (IPGTT) from 12,742 ± 1113 at T0 to 8580 ± 1132 after 4 weeks of Aerobic Training (*p* < 0.001) [7]. These findings support the role of physical activity in improving glucose tolerance. In contrast, if the body switches to an inactive or sedentary lifestyle, all the physiological and metabolic adaptations gained through exercise could be lost or reversed. This process, known as detraining, eventually leads to reduced insulin sensitivity and disruption of glucose homeostasis [8].

In addition to the metabolic activity of skeletal muscle, it has been recognized for secretory function, with the production and release of a large variety of cytokines and other peptides known as myokines [9]. Myokines regulate glucose metabolism and insulin secretion [10,11]. Interleukin (IL)-6, insulin-like growth factor-1 (IGF-1), fibroblast growth factor-21 (FGF-21), myostatin, and irisin [12] are among the several hundred secretory products that constitute the skeletal muscle secretome. Exercise training positively modulates myokine secretion by decreasing myostatin and increasing irisin, IL-6, FGF-21, and IGF-1 levels. These changes contribute to the regulation of energy expenditure, insulin sensitivity, and glucose homeostasis both locally within the muscle and systematically in other organs. These exercise-induced myokines have the potential to manage T2D [13]. For instance, the reduction in myostatin levels during moderate Aerobic Training is associated with improved insulin sensitivity [14] and elevated expression of GLUT-1 and GLUT-4 in rat muscle [15], thus improving glucose tolerance.

Moreover, physical activity stimulates IL-6 secretion, which plays a crucial role in the regulation of glucose metabolism [16]. In response to exercise, various other proteins are secreted by the skeletal muscles, such as FGF-21 and irisin [17,18,19]. In skeletal muscle, training-induced irisin regulates glucose metabolism by upregulating the AMPK pathway and GLUT-4 translocation [20], whereas FGF-21 enhances glucose uptake, GLUT-1, and phosphoinositide-3-kinase–protein kinase/Akt1 (PI3K/Akt1) [21,22], thereby preventing T2D. In turn, detraining may negatively alter myokine secretion and disrupt glucose metabolism, because most of the beneficial effects on myokine secretion result from muscle activity or contraction. To the best of our knowledge, data on the effects of detraining on myokines are limited. Therefore, it would be interesting to determine how myokine secretion is affected when the body shifts from training to detraining and to identify the metabolic changes associated with it.

In addition to exercise, diet is also a critical component of health. The fundamental relationship between diet, health, and disease has diverted researchers’ attention toward phenolic compounds, which are secondary plant metabolites found in fruits and vegetables. Phenolic compounds, such as Protocatechuic Acid (PCA), have shown potential in regulating insulin-signaling pathways, such as IRS-1 and PI3K-Akt [23], and in increasing GLUT-4 translocation and glucose uptake [24], thus preventing and treating T2D [25]. In addition, PCA can enhance antioxidant capacity [26] and preserve skeletal muscle strength and endurance [27]. Some phenolic compounds have been shown to positively modulate myokine secretions. For instance, FGF-21 levels increased with cyanidin-3-glucoside supplementation in an in vitro model [28] and with quercetin dosage in an animal study [29]. Additionally, the administration of phenolic-rich olive leaf extract and caffeic acid in rats suppressed myostatin and elevated IGF-1 levels [30] and the PI3K/Akt pathway [31]. However, the role of PCA in myokines and glucose homeostasis remains unclear. To date, only one animal study has demonstrated that PCA administration regulates IGF-1 concentration [32].

Currently, there is a surge in metabolic diseases caused by excessive food availability and lack of physical activity. From an evolutionary perspective, humans have been biologically designed to be physically active [33]. However, adaptation to inactive and sedentary lifestyles began around 2002 A.D., with technological advancements further accelerating this trend. Modern societies have significantly transformed people’s lifestyles, leading to a substantial increase in physical inactivity. As evolution is a slow-acting process, modern humans have not yet fully adapted to an inactive/sedentary lifestyle [34]. About 99.5% of humans live as hunter-gatherers, and as a result, the human genetic makeup and metabolic programming remain relatively similar to those of our ancestral hunter-gatherers [35]. Consequently, there has been an increasing prevalence of metabolic diseases, including metabolic syndrome, type 2 diabetes, non-alcoholic fatty liver disease, and cardiovascular disease [36]. This phenomenon is illustrated by the example of Paleo-Indians, who, upon adopting a sedentary lifestyle with a constant food supply, developed non-insulin-dependent diabetes mellitus [37]. As a result, it has become crucial to identify shifts in metabolic processes and muscle secretions that have occurred in humankind due to the adoption of a modern lifestyle. The current study focused on identifying the changes that occur when the body shifts to detraining or inactivity, as studies on maintaining a fully trained state have already been extensively conducted [38,39].

Certainly, the individual benefits of physical activity on skeletal muscle secretion are well established. In fact, some previous findings have highlighted the combined effect of physical activity and phenolic compounds in improving elevated IGF-1 expression [40], irisin levels [41], FGF-21 concentrations [42], and blood glucose [42]. However, there is a notable lack of evidence regarding the impact of detraining alone and in combination with phenolic compounds on myokine secretion. Therefore, it would be interesting to explore the potential synergy of phenolic compounds with moderate-intensity exercise training and their counteracting effect on detraining.

To our knowledge, this is the first study to examine the combined effects of physical training and detraining with PCA supplementation on muscle secretions. Accordingly, the present study investigated the impact of moderate-intense exercise training, detraining alone or in combination with PCA supplementation on myokines and glucose metabolism.

### Aim and Objectives

We aim to study whether moderate-intensity physical training induces the same exercise-associated metabolic changes as endurance training. Hypothetically, the experimental data we achieve will provide new insight into the impact of moderate-intensity training, detraining, and supplementation of antioxidants on myokine metabolism. The study will focus on the production of myokines and the regulation of insulin-signaling pathways.

To fulfill the aim, we formulate the following objectives:

1. Model moderate-intensity physical training in male Wistar rats;
2. Explore the metabolic alterations that occur during the training period, the transition from the trained state to the detrained state, and the counteracting effect of PCA;
3. Compare the impact of training, detraining, and PCA supplementation on insulin-signaling pathways and GLUT-4 expression;
4. Assess relationships among studied variables, and find their associations reflecting the mode of training, changes in myokine metabolism, insulin-signaling mechanisms, and PCA supplementation.

## 2. Materials and Methods

### 2.1. Study Methodology

The first objective involves the design and implementation of 4 weeks of moderate-intensity physical training in male Wistar rats. On day one, the treadmill was set at 5 m/min for 5 min; the treadmill speed increased by 1 m/min daily to familiarize the rats with the treadmill during week one. After week one, the speed gradually increased until it reached 25 m/min and a duration of 60 min. This was to create a model of moderately intense trained animals. However, animals underwent 4 weeks of training followed by 8 weeks of detraining and 8 weeks of training followed by 4 weeks of detraining. During the detrained period, animals stopped running on the treadmill. This was to compare the effects of varying levels (4 weeks and 8 weeks) of exercise training and detraining. Moreover, we assessed how detraining may lead to loss of training-induced effects. This helped us to explore the ideal minimum duration (either 4 or 8 weeks) required to induce positive alteration in myokines and metabolic outcomes. Similarly, the duration of detraining is enough to reverse all positive alterations induced by training.

The second objective focuses on the metabolic changes during different training and detraining durations. We investigated the role of antioxidant supplementation, particularly PCA, in combination with training and detraining periods. This aspect was to determine the synergistic role of exercise and PCA. We also studied the potential counteracting effect of PCA during detraining. Working on the second objective, we analyzed muscle secretions in the gastrocnemius muscle. This helped us to identify the changes in muscle secretions during training and detraining with or without PCA supplementation. The data on myokines (IGF-1, IL-6, FGF-21, myostatin, irisin) were checked for accuracy. Two-way analysis of variance (ANOVA) with a Sidak post hoc test and pairwise comparisons were performed to assess the main effects of time periods, groups, and any potential interaction effects.

To address the third objective, insulin-signaling pathways were studied using the Western blot technique. Then, the expression of PI3K, IRS-1, IRS-2, GLUT-4, and P-Akt/Akt ratio was compared among the groups with the ANOVA test. A pairwise comparison was performed using the Tukey test to determine the differences among various treatments.

The fourth objective was two-fold. In the first part of objective four, we preprocessed the data in the mean normalization technique. To fulfill missing values, we resorted to linear imputations. After data preprocessing, we conducted a factor analysis to describe variability among observed, correlated data. In particular, we used principal component analysis and linearly transformed the data matrix onto a new coordinate system in the Varimax rotation technique. Eigenvalues of 1.0 or greater were taken into analysis. Absolute loadings greater than 0.755, from 0.75 to 0.50, and below 0.50 were considered ‘strong’, ‘moderate’, and ‘weak’, respectively. This approach to data analysis allowed for identifying the directions and capturing the largest variation in the data.

In the second part of objective four, we resorted to unsupervised and supervised learning methods. In an unsupervised technique, we explored the naturally occurring groups within the study dataset. We took the following three groups of data into the cluster analysis: the duration of training and detraining, the level of myokines, and the expression of proteins. With clustering, we tried to highlight the cross-variable dependencies that remained hidden until we performed this analysis.

To find the distinctions between the studied groups of animals, we implemented discriminant analysis. It helped us to find the independent variables that effectively separate the group of animals treated with and without PCA supplementation. In this analysis, the concentration of myokines and protein expression were used as predictors to identify the treatment effect.

### 2.2. Sample Size Calculations and Animal Care

Mead’s Resource Equation was used to determine the sample size of the animal study [43]. According to this equation, two to three animals per group are required. We followed the ethical principles of the 3Rs (Replacement, Reduction, and Refinement) to carefully choose the animals and sample size. Therefore, the total number of animals used in this study was forty-seven.

The reporting of animal data in this study adhered to the ARRIVE guidelines. Animal care and all related procedures were conducted in accordance with the ethical treatment and use guidelines for animals in research established by the National Institutes of Health (NIH), USA.

### 2.3. Protocatechuic Acid Supplementation

A 97% pure Protocatechuic Acid (PCA) (Sigma-Aldrich, MO, USA) was administered at a dose of 100 mg/kg/day for 8 weeks (from 4 to 12 weeks) via oral gavage. PCA at a dose of 100 mg/kg showed strong efficacy [44]. Previously, a greater potential of PCA at a dose of 100 mg/kg body weight was observed to improve various metabolic biomarkers, including lipid profile, glucose level, and insulin level [45]. Therefore, PCA at a dose of 100 mg/kg body weight was used in the present study. PCA was dissolved in 2 mL of water with 0.5% carboxymethylcellulose (CMC) (Sigma-Aldrich, MO, USA) as a vehicle.

### 2.4. Determination of Myokines

Gastrocnemius muscles were homogenized with cold lysis Kcl buffer (10 mM Tris base, pH 8, NaCl (140 mM), KCl (300 mM), 0.5% SDC, EDTA (1 mM), 0.5% sodium deoxycholate and 0.5% Triton X-100) with addition of 1% protease phosphatase inhibitor cocktail (Sigma Aldrich, St. Louis, MO, USA), using a rotor–stator homogenizer. Tissue homogenates were centrifuged at 4 °C with a speed of 15,000 g, for 25 min, and stored the supernatants at −80 °C.

Myokines, including IGF-1, IL-6, FGF-21, myostatin, and irisin, were assessed in gastrocnemius muscle homogenates using enzyme-linked immunosorbent assay (ELISA), and manufacturer’s instructions were followed to perform ELISA analysis. For IGF-1 (Cat. # MG100, R&D system, Minneapolis, MN, USA) and myostatin (Cat. # DGDF80, R&D system, Minneapolis, MN, USA) measurements, an ELISA kit with sensitivities of 3.5 pg/mL and 2.25 pg/mL, respectively, were used. The IL-6 level was measured by an Invitrogen kit (Cat. # ERA32RB, Thermo Fisher Scientific, Waltham, MA, USA) with a sensitivity of 15 pg/mL. Myokines, such as FGF-21 and irisin, were measured in muscle homogenates using MyBioSource Kits (Cat. # MBS2024083 and Cat. # MBS7205414, San Diego, CA, USA) with a sensitivity of 5.9 pg/mL and 0.1 µg/mL, respectively.

### 2.5. Muscle Protein and Insulin-Signaling Pathways Analysis

The expressions of insulin receptors (IRs) IRS-1, IRS-2, PI3K, Akt, P-Akt, and GLUT-4 were analyzed in gastrocnemius muscle homogenates. Total protein was extracted from the gastrocnemius muscle in RIPA lysis buffer (Sigma Aldrich, MO, USA) containing a 1% protease phosphatase inhibitor cocktail (Sigma Aldrich, MO, USA), with a tissue homogenizer. Protein concentration in the supernatant was assessed by the BCA Protein Assay Kit (Sigma Aldrich, MO, USA). Afterward, the supernatant was diluted in 6 × RIPA buffer and incubated at 90 °C for 5 min.

A total of 35 μg protein per sample was loaded into each well and subjected to SDS–PAGE. Then, the gel was transferred onto a nitrocellulose membrane using a Bio-Rad electro-transfer apparatus. Blocking of membranes was performed with 5% non-fat milk in Tris-buffered saline containing 0.1% Tween-20 (TBS-T) for one hour at room temperature. Primary antibodies, including GLUT-4 (1F8), PI3K (p85), Akt, P-Akt (S473), IRS-1, and IRS-2 (Cell Signaling Technology, Danvers, MA, USA), were applied to incubate the membrane overnight. After three washes with TBS-T, the membranes were incubated for 1 h with horseradish peroxidase-conjugated secondary antibodies (anti-rabbit IgG and anti-mouse IgG; Jackson Immune Research, Cambridge House, UK). Bands were detected using an enhanced chemiluminescence detection kit (Bio-Rad, Hercules, CA, USA), and quantification of band densities was conducted using the Quantity One System image analyzer (Bio-Rad, Hercules, CA, USA).

## 3. Results

### 3.1. Modeling Moderate-Intensity Physical Training in Male Wistar Rats

At baseline (T0), six rats were euthanized by decapitation. The remaining 41 rats have gone through a 4-week treadmill exercise training program (six lanes; Columbus Instruments Treadmill Simplex II, Columbus, OH, USA). The exercise training protocol and animal maintenance were performed as described in our previous study (14). Lactate levels were assessed while the rats were running at five-time points (0, 5, 10, 15, and 25 min) at running speeds of 15, 20, 25, and 30 m/min to measure exercise capacity (Supplementary Figure S1A–C). A previous review by Wang et al. categorized the intensity of treadmill exercise based on running speed and treadmill slope into low intensity (<18 m/min with 0–5% slope), moderate intensity (18–25 m/min with 0–10% slope), and high intensity (>25 m/min with ≥0% slope) [46].

**Figure 1:**
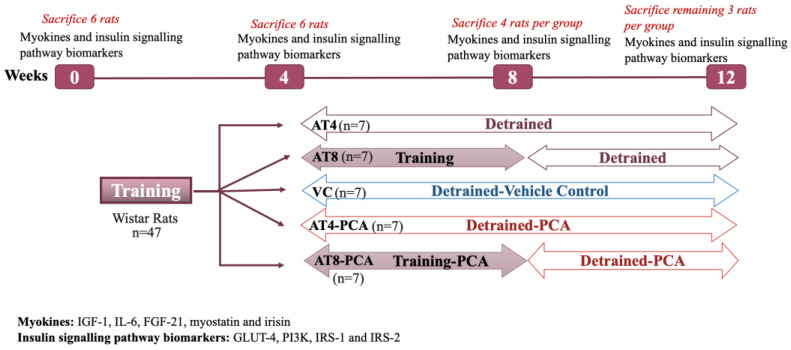
Study protocol. Aerobic Training of 4 weeks (AT4), Aerobic Training of 8 weeks (AT8), Vehicle Control (VC), Aerobic Training of 4 weeks + Protocatechuic Acid (AT4-PCA), Aerobic Training of 8 weeks + Protocatechuic Acid (AT8-PCA).

After 4 weeks of training (T4), the decapitation method was used to euthanize six rats, 48 h after the last exercise session to eliminate any acute effects of exercise. The remaining 35 rats were randomly assigned to one of the five groups: (1) Aerobic Training for 4 weeks (AT4), then 8 weeks of detraining; (2) Aerobic Training for 8 weeks (AT8)—the group was assigned to run on a treadmill at a speed of 25 m/min for 60 min per day, for 5 days in a week, for a total of 8 weeks, then 4 weeks of detraining; (3) Vehicle Control (VC) detrained after week 4 and only received carboxymethyl cellulose in water (CMC) from T4 to T12; (4) Aerobic Training for 4 weeks, then no training but a daily dose of PCA (AT4-PCA); (5) Aerobic Training for 8 weeks, then no training and daily dose of PCA from T4 to T12 (AT8-PCA). After 8 weeks, four rats per group were sacrificed by decapitation. At T12, all the remaining animals (three rats per group) were sacrificed by decapitation (Figure 1). After sacrifice, the gastrocnemius muscle was harvested, cleaned of fat tissue, flash-frozen in liquid nitrogen, and stored at −80 °C.

### 3.2. Muscle Secretions

#### 3.2.1. Insulin-like Growth Factor-1 (IGF-1)

**Figure 2:**
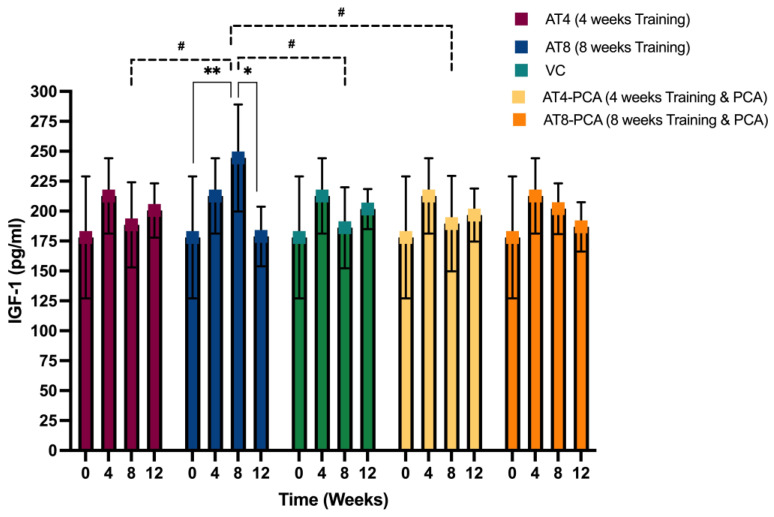
The concentration of insulin-like growth factor-1 (IGF-1) in rats gastrocnemius muscle tissue (pg/mL). Values are presented as mean ± standard deviation (S.D) of the duplicate experiment. Statistical significance was set at *p* ≤ 0.05. Statistically significant difference among groups * *p* ≤ 0.05, ** *p* ≤ 0.01. Statistically significant difference among time points # *p* ≤ 0.05. Aerobic Training of 4 weeks (AT4), Aerobic Training of 8 weeks (AT8), Vehicle Control (VC), Aerobic Training of 4 weeks + Protocatechuic Acid (AT4-PCA), Aerobic Training of 8 weeks + Protocatechuic Acid (AT8-PCA). Time points: week 0, week 4, week 8, week 12.

There was a significant effect of exercise training on IGF-1 levels, across time points [F (3, 168) = 8.343, *p* < 0.001], as revealed by a two-way ANOVA. No significant interaction was found between time points and groups [F (12, 168) = 1.067, *p* = 0.391]. Regular moderate-intense exercise training for 4 weeks increased IGF-1 level from 178.03 ± 50.93 pg/mL at T0 to 212.64 ± 31.44 pg/mL at T4 (Figure 2). While 8 weeks of training (AT8) further increased IGF-1 to 244.42 ± 44.71 pg/mL (*p* < 0.007) at T8, significantly different from the value at T0. However, after 4 weeks of detraining, in AT4 at T8, the IGF-1 level declined to 188.48 ± 35.57 pg/mL, and no significant change was observed at T12. In contrast, in AT8, at T12, the IGF-1 level decreased significantly compared to that at T8 and returned to a level similar to that at T0. The trends in groups AT4-PCA (4 weeks of training with PCA) and VC were similar to those observed in AT4 (only 4 weeks of training without PCA).

There was a significant difference in IGF-1 level with 8 weeks of training (AT8) compared to 4 weeks of training (AT4, VC, AT4-PCA) at T8. However, no significant change was seen with PCA supplements in the group that received 8 weeks of training with PCA dose (AT8-PCA) compared to the group that received only 8 weeks of training without PCA (AT8).

#### 3.2.2. Interleukin (IL)-6

**Figure 3:**
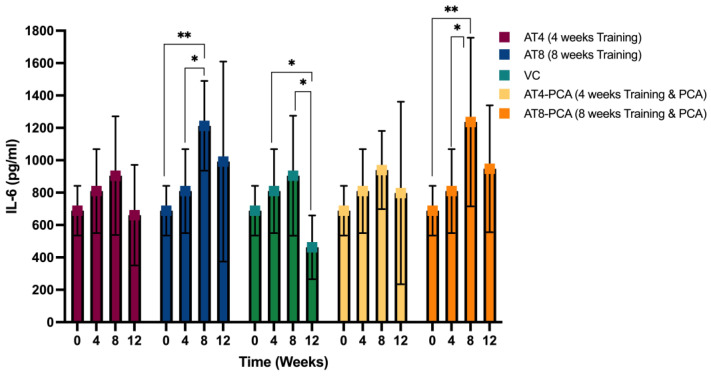
The concentration of Interleukin (IL)-6, in rats gastrocnemius muscle tissue (pg/mL). Values are presented as mean ± S.D of the duplicate experiment. Statistical significance was set at *p* ≤ 0.05. Statistically significant difference among groups * *p* ≤ 0.05, ** *p* ≤ 0.01. Aerobic Training of 4 weeks (AT4), Aerobic Training of 8 weeks (AT8), Vehicle Control (VC), Aerobic Training of 4 weeks + Protocatechuic Acid (AT4-PCA), Aerobic Training of 8 weeks + Protocatechuic Acid (AT8-PCA). Time points: week 0, week 4, week 8, week 12.

Two-way ANOVA revealed a significant effect of exercise training on IL-6 levels across time points [F (3, 161) = 10.774, *p* < 0.000] and groups [F (4, 161) = 3.307, *p* < 0.012]. No significant interaction was found between the time points and groups [F (12, 161) = 1.260, *p* = 0.247]. Similar trends were observed in all groups: IL-6 levels tended to increase continuously from T0 to T8 and then decreased at T12 (Figure 3). The increase in IL-6 levels at T4 (with 4 weeks of training) was not significant, while it was significant when training continued for 8 weeks (at T8) in AT8 and AT8-PCA (*p* < 0.05). Subsequently, IL-6 levels reached 1212.76 ± 277.35 pg/mL in AT8 (8 weeks of training without PCA) and 1236.00 ± 520.67 pg/mL (*p* < 0.05) in AT8-PCA (8 weeks of training with PCA). Compared to the group that only trained for 4 weeks (AT4), the IL-6 level was 904.84 ± 3.66.58 pg/mL at T8.

The IL-6 levels dropped to 660.98 ± 310.44 pg/mL in the AT4 group after 8 weeks of detraining compared to the group that was only detrained for 4 weeks (AT8) (991.74 ± 617.77 pg/mL) at T12. Similarly, 8 weeks of detraining reduced IL-6 level to 797.64 ± 563.63 pg/mL in AT4-PCA compared to 4 weeks of detraining in AT8-PCA (947.64 ± 391.74 pg/mL). PCA supplementation did not show any positive impact on detraining. However, the result was significant only in the VC group after 8 weeks of detraining compared to T4 and T8 time points (*p* < 0.05). We did not find any significant differences between the groups at any time point.

#### 3.2.3. Fibroblast Growth Factor-21 (FGF-21)

**Figure 4:**
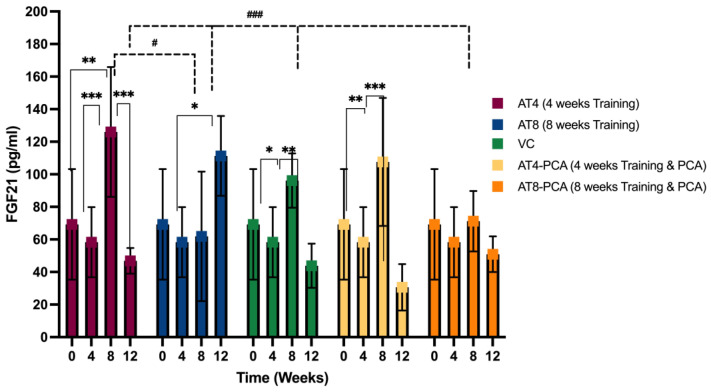
The concentration of fibroblast growth factor-21 (FGF-21) in rats gastrocnemius muscle tissue (pg/mL). Values are presented as mean ± S.D of the duplicate experiment. Statistical significance was set at *p* ≤ 0.05. Statistically significant difference among groups * *p* ≤ 0.05, ** *p* ≤ 0.01, *** *p* ≤ 0.001. Statistically significant difference among time points # *p* ≤ 0.05, ### *p* ≤ 0.001. Aerobic Training of 4 weeks (AT4), Aerobic Training of 8 weeks (AT8), Vehicle Control (VC), Aerobic Training of 4 weeks + Protocatechuic Acid (AT4-PCA), Aerobic Training of 8 weeks + Protocatechuic Acid (AT8-PCA). Time points: week 0, week 4, week 8, week 12.

Exercise training had a significant impact on FGF-21 levels across time points [F (3, 149) = 12.702, *p* < 0.000], as revealed by the two-way ANOVA. There was a significant interaction between the time points and groups [F (12, 149) = 3.662, *p* = 0.000]. At T4, none of the groups showed significant changes in the FGF-21 levels. The groups only trained for 4 weeks, such as AT4, VC, and AT4-PCA; when detrained for 4 weeks, they showed a significant (*p* < 0.05) elevation in FGF-21 level by 126.03 ± 39.87 pg/mL, 96.11 ± 16.67 pg/mL, and 71.23 ± 18.55 pg/mL, respectively, at T8 (Figure 4). Comparatively, no change was observed in FGF-21 levels in the AT8 and AT8-PCA groups who continued training for 8 weeks at T8.

A similar trend of increasing FGF-21 levels was seen in AT8 (111.30 ± 24.49 pg/mL (*p* = 0.029)) after training cessation for 4 weeks, at T12, showing that FGF-21 level increased during detraining. However, this was not observed in the AT8-PCA group who received PCA supplementation with 8 weeks of training. In addition, there was a significant difference between 4-week training (AT4) and 8 weeks of training (AT8) at T8 (*p* = 0.021). Group AT8 (8 weeks of training without PCA) showed a significant difference from all other groups (AT4, VC, AT4-PCA, and AT8-PCA) (*p* < 0.001) at T12 (Figure 4).

#### 3.2.4. Myostatin

**Figure 5:**
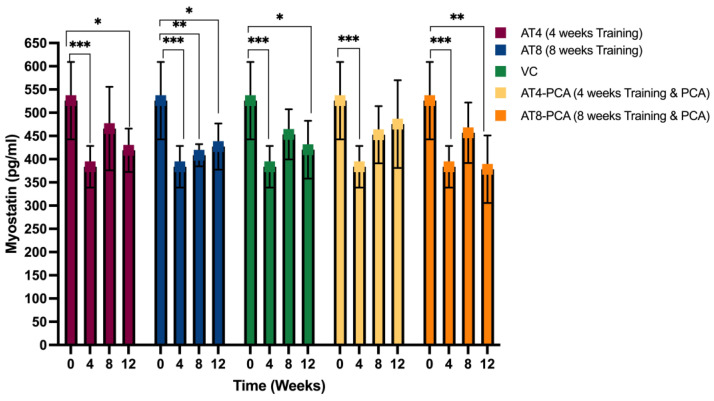
The concentration of myostatin in rat gastrocnemius muscle tissue (pg/mL). Values are presented as mean ± S.D of the duplicate experiment. Statistical significance was set at *p* ≤ 0.05. Statistically significant difference among groups * *p* ≤ 0.05, ** *p* ≤ 0.01, *** *p* ≤ 0.001. Aerobic Training of 4 weeks (AT4), Aerobic Training of 8 weeks (AT8), Vehicle Control (VC), Aerobic Training of 4 weeks + Protocatechuic Acid (AT4-PCA), Aerobic Training of 8 weeks + Protocatechuic Acid (AT8-PCA). Time points: week 0, week 4, week 8, week 12.

Exercise training had a significant effect on myostatin levels across time points [F (3, 164) = 48.021, *p* < 0.000]. No significant interaction was found between time points and groups [F (12, 164) = 0.706, *p* = 0.744]. Training for 4 weeks significantly decreased myostatin level from 525.97 ± 83.39 pg/mL at T0 to 383.65 ± 44.73 pg/mL at T4 (*p* < 0.001) (Figure 5). The groups that received only 4-week training (AT4, AT4-PCA, and VC) reported having enhanced myostatin levels after 4 weeks of detraining at T8, but this was not significant. The groups received 8 weeks of training (AT8 and AT8); the levels of myostatin remained significantly lower in those groups compared to baseline (T0). Even after detraining at T12, myostatin level remained lower by 427.13 ± 49.51 pg/mL in AT8 and 378.25 ± 72.69 pg/mL in AT8-PCA (*p* < 0.01). There were no significant differences among groups at any time point.

#### 3.2.5. Irisin

**Figure 6:**
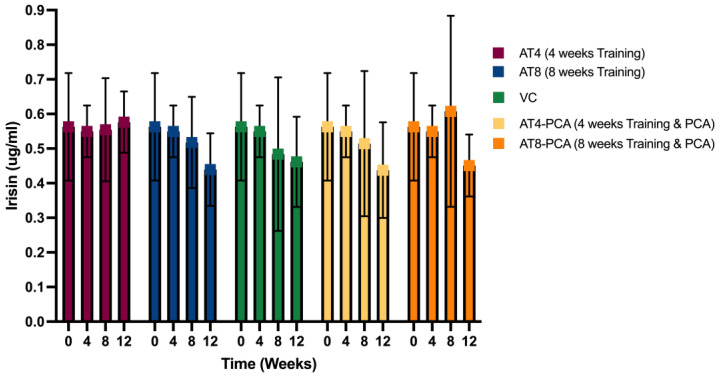
The concentration of Irisin in rat gastrocnemius muscle tissue (µg/mL). Values are presented as mean ± S.D of the duplicate experiment. Statistical significance was set at *p* ≤ 0.05, Aerobic Training of 4 weeks (AT4), Aerobic Training of 8 weeks (AT8), Vehicle Control (VC), Aerobic Training of 4 weeks + Protocatechuic Acid (AT4-PCA), Aerobic Training of 8 weeks + Protocatechuic Acid (AT8-PCA). Time points: week 0, week 4, week 8, week 12.

No significant differences were detected between the time points and between the groups. There was no significant interaction between the time points and groups [F (12, 93) = 0.335, *p* = 0.981] (Figure 6).

### 3.3. Protein Expressions

**Figure 7:**
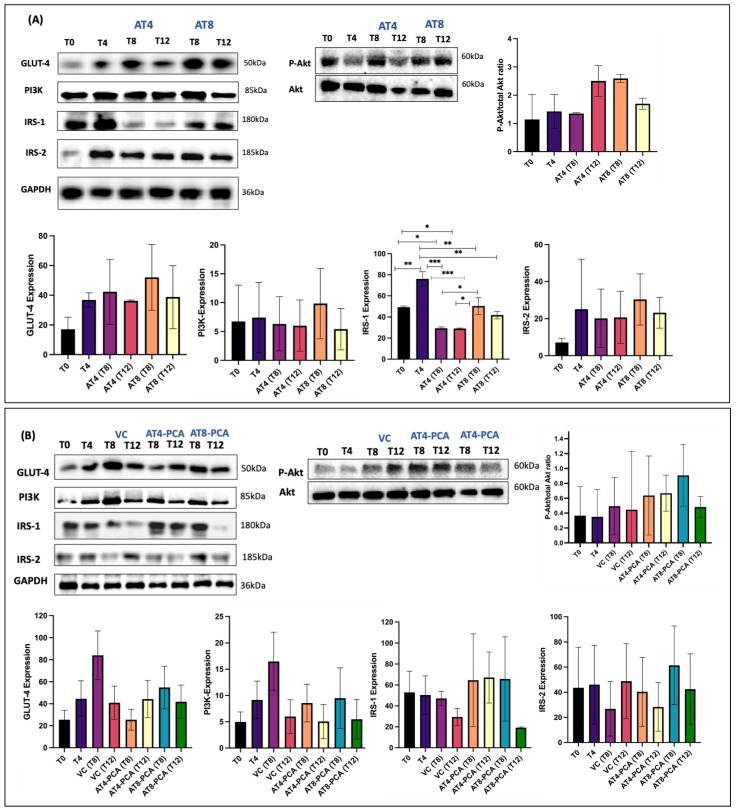
Glucose transporter-4 (GLUT-4), phosphatidylinositol (PI) 3-kinase (PI3K), insulin receptor substrate (IRS) -1 (IRS-1), IRS-2, Akt and P-Akt protein expression. (**A**) Groups: Aerobic Training of 4 weeks (AT4), Aerobic Training of 8 weeks (AT8). (**B**) Groups: Vehicle Control (VC), Aerobic Training of 4 weeks + Protocatechuic Acid (AT4-PCA), Aerobic Training of 8 weeks + Protocatechuic Acid (AT8-PCA). Time points: week 0 (T0), week 4 (T4), week 8 (T8), week 12 (T12). Values are the mean ± S.D. of the duplicate experiment. ANOVA with multiple comparisons was performed and statistical significance was set at *p* ≤ 0.05. Statistically significant * *p* ≤ 0.05, ** *p* ≤ 0.01, *** *p* ≤ 0.001.

GLUT-4 expression increased by 1.1-fold after 4 weeks of training and continued to increase in AT8 after 8 weeks of training at T8 by 0.4-fold compared to T4 (Figure 7A). Training for 8 weeks (AT8) improved the GLUT-4 expression by 0.2-fold compared to 4 weeks training (AT4) at T8. However, in AT4-PCA at T8, there was a decrease in GLUT-4 expression after 4 weeks of detraining, but at T12 with PCA supplementation, GLUT-4 increased by 0.7-fold compared to T8. A similar trend was observed in AT8-PCA (trained for 8 weeks with PCA supplementation) in which GLUT-4 expression increased by 1-fold at T8 compared to that at T4 (Figure 7B).

PI3K expression slightly increased after 4 weeks of training by 0.1-fold. In AT8, after 8 weeks of training, at T8, PI3K and Akt expression were elevated by 0.3-fold and 0.04-fold, respectively, compared to T4. In AT8 (8 weeks of training), PI3K increased by 0.6-fold compared to AT4 (4 weeks of training) at T8. PI3K expression decreased after 4 weeks of detraining at T8, in AT4 by 0.1-fold, and in AT4-PCA by 0.4-fold. Detraining at T12 reduced PI3K expression in AT8 by 0.4-fold and AT8-PCA by 0.5-fold. However, PCA supplementation in AT4-PCA and AT8-PCA groups enhanced P-Akt/Akt ratio by 0.4-fold and 1.5-fold, respectively, at T8.

Training for four weeks did not cause any changes in IRS-1 expression. However, 8-week training alone (AT8) and in combination with PCA (AT8-PCA) increased IRS-1 expression by 0.8-fold and 0.3-fold at T8, respectively, compared to T4. Notably, PCA supplementation attenuated the negative effect of detraining in AT4-PCA at T8 and T12; however, this positive effect of PCA on detraining was not observed in AT8-PCA at T12. An increase in IRS-2 was observed in AT8 and AT8-PCA by 0.2-fold and 0.3-fold, respectively, with 8-week training compared with T4. Detraining reduced IRS-2 expression in all the groups.

### 3.4. Data Analysis

The varimax rotated six-factor matrix is presented in Table 1. PCA supplementation has the highest factor loading of 0.97, which describes it as the most important variable in the dataset. Drug supplementation can explain the important processes taking place in the medium. The absolute loadings of IGF-1 and myostatin are high on the second factor: 0.74 and −0.78, respectively. This factor describes the level of myokines secreted due to the physical load. Group number has a large positive loading on factor 3. It accounts for training settings; therefore, its role in the data variance is high. The loadings on factors 5 and 6 are high for the level of IL-6 and the expression of GLUT-4, respectively. Given this analysis, we can label these factors ‘immune status factor’ and ‘factor of glucose transport in skeletal muscles.

**Figure 8:**
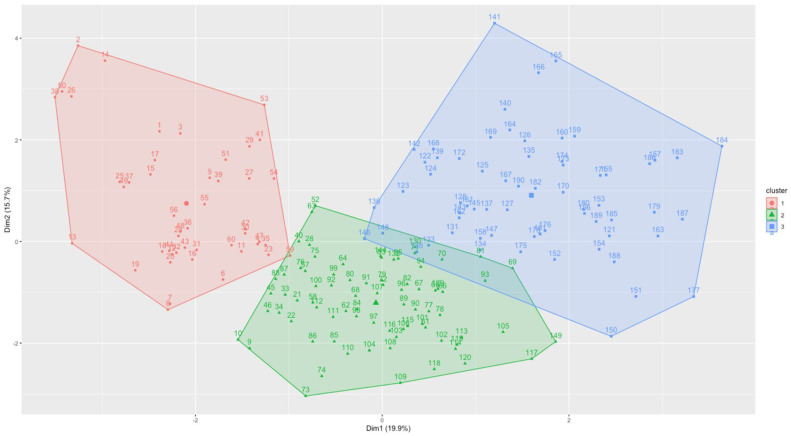
Cluster plots show high separability of data.

To determine the optimal number of clusters, we used statistical testing methods (gap statistics) and direct methods (within-cluster sums of squares and the average silhouette and within-cluster sums of squares with the elbow rule). Most of these methods showed the optimal number of three clusters. The results of the clustering analysis demonstrated the distinct separability of clusters that correspond to the duration of the training and PCA supplementation. The clusters were differentiated by the duration of exercise training and the administration of PCA supplementation, with cluster one representing 8 weeks of training, cluster 2 representing 4 weeks of training without detraining, and the third representing those who received PCA supplementation. This separation highlights the influence of both exercise duration and PCA supplementation on the observed metabolic markers (see Figure 8, Table 2).

With the discriminant analysis, we tried to classify the animals into two groups: those who were administered PCA and those who did not receive the drug. As seen in Table 3, the most important predictors were the levels of FGF-21, IL-6, and the expression of IRS-1. The total accuracy of the classification model was high: 86.8%. The animals treated without PCA were identified more accurately due to data disbalance. 

## 4. Discussion

Exercise stimulates the myokine secretions within skeletal muscles and increases their levels in circulation, leading to a significant overall elevation in response to physical activity. For instance, an acute bout of moderate-intensity cycling on a stationary bicycle elevated the expression of IL-6 and FGF-21, but the serum levels of IL-6, IL-15, and FGF-21 increased significantly [47]. Indeed, exercise has an impact on the local secretion of myokines from skeletal muscle, but this does not always correspond to their release into the circulation. Certain myokines are believed to act directly on skeletal muscles, thereby enhancing energy metabolism during contraction [48]. Therefore, analyzing myokines directly in skeletal muscle is crucial for accurately understanding their role, as serum measurements can be influenced by other systemic factors.

In this context, the present study investigated the impact of moderate-intensity exercise training and detraining on myokines and insulin-signaling pathways in gastrocnemius muscle. We also explored the potential impact of PCA with physical training and detraining. Physical training elevated IGF-1 and IL-6 levels and reduced myostatin and FGF-21 levels. These training-induced myokines result in increased GLUT-4 expression and activation of insulin-signaling pathways such as PI3K, P-Akt/Akt, IRS-1, and IRS-2 expression. Additionally, PCA attenuated the negative impact of detraining by modulating myokine secretions, insulin-signaling pathways, and GLUT-4 expression.

Exercise training beneficially modulates myokines (IL-6, IGF-1, FGF-21, myostatin, and irisin), while detraining may reduce or reverse these positive effects [49]. Insulin-like growth factor-1 (IGF-1) is a myokine stimulated by physical training, which helps improve insulin sensitivity and regulates blood glucose. IGF-I can stimulate glucose transport activity in skeletal muscles by activating the PI3K pathway and translocating GLUT-4 [50,51]. Nonetheless, exercise increases the expression of IGF-1, which may be a distinct regulator of the PI3K pathway and GLUT-4 translocation [52]. Evidence showed that one session of the moderate-intensity treadmill and ergo-cycle exercise for 30 min in obese females elevated IGF-1 [51]. Similarly, we noted a 19% increase in IGF-1 level after 4 weeks of training, and when training continued for 8 weeks, the IGF-1 level increased by 14% in the AT8 group compared to T4. The group AT8-PCA was trained for 8 weeks with PCA and showed a 6% increase in IGF-1 compared to the group that was only trained for 4 weeks but received the PCA dose (AT4-PCA) at T8. However, a 26% decline was observed after 4 weeks of detraining in AT8 at T12 compared to T8 (*p* < 0.03).

It is well established that skeletal muscle synthesizes and releases large amounts of interleukin-6 (IL-6) in response to physical activity. IL-6 was discovered to be a myokine when a reported increase in IL-6 by 100-fold in response to exercise demonstrated substantial production compared with other cytokines. Exercise stimulates IL-6 production locally but also induces hepatic glucose output, implying a crucial link between muscle contraction and metabolic alterations mediated by IL-6 [53]. Interestingly, increased IL-6 levels during exercise show no sign of muscle damage, but IL-6, as a myokine, plays an important role in regulating glucose metabolism [54,55]. Correspondingly, we observed an 18% increase in IL-6 concentration after 4 weeks of training and further increased after 8 weeks of training in AT8 and AT8-PCA by 34% and 32%, respectively, compared to AT4 and AT4-PCA. Our results are consistent with Isanejad et al. findings, which demonstrated that endurance training significantly increased IL-6 in both the soleus and extensor digitorum longus muscles (*p* < 0.05) [56]. Moreover, human studies have shown increased IL-6 levels during exercise, including acute sprint exercise [57] and high-intensity interval exercise [58]. Nevertheless, the IL-6 gene remains silent in resting muscle but is promptly activated due to muscle contraction [59]. Likewise, we reported that detraining resulted in a drastic decline in IL-6 levels at T12 in AT8 by 18% and AT8-PCA by 23% in rats.

Exercise-induced muscle contraction positively modulates the secretion of fibroblast growth factor-21 (FGF-21) in skeletal muscles. Muscular FGF-21 enhances glucose uptake, increases GLUT-1 and GLUT-4 expressions, and improves insulin sensitivity [21,22]. However, the present study reported a reduction in FGF-21 levels in response to exercise in all groups. This is consistent with previous findings demonstrating that 12 weeks of resistance training significantly decreased FGF-21 and myostatin levels in elderly men with and without T2D [19]. Taniguchi et al. demonstrated that a 5-week endurance exercise program reduced serum FGF-21 concentration and hepatic fat content in elderly men. The authors suggested that prolonged exercise might reduce FGF-21 resistance, similar to insulin resistance, thereby decreasing the FGF-21 level [60]. Similarly, our data showed a reduction in FGF-21 levels with exercise training.

However, detraining significantly increased the FGF-21 levels at T8 in the groups that were trained for 4 weeks (AT4, VC, and AT4-PCA). Comparatively, in the group that continued training for 8 weeks (AT8), the level of FGF-21 was maintained at T8 but increased abruptly at T12 after four weeks of detraining. Regular training may modulate FGF-21 secretion, thereby improving FGF-21 resistance. Detraining could result in a reduction in FGF-21 resistance, thus enhancing FGF-21 levels. On the other hand, FGF-21 levels were maintained even after training cessation at T12 in the group trained for 8 weeks and received PCA supplementation (AT8-PCA), suggesting that PCA may have attenuated the negative effects of detraining at T12, which is in line with previous findings showing that dietary curcumin attenuated high-fat diet-induced FGF-21 resistance [61]. Similarly, another study reported a reduction in FGF-21 mRNA and FGF-21 levels in serum with resveratrol supplementation and high- and moderate-intensity exercise training in rats [62].

Myostatin plays an important role in skeletal muscle growth and metabolic homeostasis. Inactivation of the myostatin gene in mice improved whole-body insulin sensitivity, glucose uptake, and GLUT-4 expression [63]. Moderate aerobic exercise reduces muscle and plasma myostatin levels and improves insulin sensitivity. Our results showed a 27% reduction in myostatin levels after 4 weeks of training at T4 compared to T0. The AT8 group that continued training for 8 weeks seemed to have a 12% lower myostatin level at T8 than the AT4 group that stopped training at T4. Our results are in accordance with the findings of Ko et al., who demonstrated that treadmill exercise for 30 min/day for six weeks suppressed myostatin mRNA and protein expression in the gastrocnemius muscle of rats [64]. Another study showed that aerobic exercise reduced myostatin protein expression in the muscle by 37% [14]. On the other hand, we noted that the myostatin levels slightly increased after 4 weeks of detraining in the AT4, VC, and AT4-PCA groups at T8. This is similar to previous findings in humans that reported that detraining increased the myostatin levels in serum [65] and myostatin mRNA levels in skeletal muscle [66].

Further, we reported that the group who received only 8 weeks of training (AT8) had slightly increased myostatin concentration at T12 after 4 weeks of detraining. In contrast, the myostatin level in the AT8-PCA group (trained for 8 weeks with PCA supplementation) was further reduced even after training cessation at T12. This might be because of PCA supplementation, which could help regulate myostatin levels even after training cessation. Similarly, evidence has shown an additional positive effect of polyphenols with training. It was found that resistance training plus epicatechin (a polyphenol) for 8 weeks reduced myostatin levels by 49% compared to resistance training alone in older people with sarcopenia. There were no significant differences among groups [67], which is similar to our findings. Indeed, the combined effect of phenolic compound and training offers enhanced benefits.

Irisin enhances glucose-stimulated insulin secretion in β-cells [17] and is believed to be modulated by exercise [68]. Our study did not report any significant differences in the irisin levels after training. In the AT8-PCA group, irisin increased at T8 after 8 weeks of training and PCA supplementation; then, there was a decline, but this did not reach statistical significance. Previous studies have also shown that serum irisin levels remain stable after acute exercise or endurance training in rats [69].

Nevertheless, physical training positively modulates myokines, which can cause metabolic alterations and improve glucose metabolism. Exercise induces the activation of insulin receptors (IRs) and phosphorylation of insulin receptor substrate (IRS), which in turn binds to the PI3K protein. Activation of PI3K/Akt results in the translocation of GLUT-4, thus increasing glucose uptake to cover the energy cost of exercise [4]. Physical inactivity can cause negative alterations in the PI3K-Akt signaling pathway, eventually leading to insulin resistance, diabetes, and metabolic diseases. In this study, 8 weeks of moderate-intense training tended to increase PI3K expression in AT8 and AT8-PCA by 36% and 23%, respectively, compared to T4. However, only 4 weeks of detraining is enough to reduce the PI3K expression. Expression of IRS-1 and IRS-2 elevated after 8 weeks of training, suggesting that the time and cumulation of exercise might be needed for their effective regulation. While detraining reduced IRS-1 and IRS-2 expressions in all groups. However, PCA supplementation for 8 weeks in AT4-PCA maintained the IRS-1 expression and IGF-1 level, but no effect was seen on IRS-2. Indeed, both IRS-1 and IRS-2 are members of the IRS protein family, but they both possess distinct regulations and functions. It might be because IRS-1 has a prominent role in skeletal muscle for insulin-dependent glucose uptake and metabolism. Contrarily, IRS-2 has shown a negligible role in insulin-induced glucose uptake in skeletal muscle [70].

We reported that GLUT-4 expression increased by 114% after 4 weeks of training and continued to increase by 41% with 8-week training in AT8 at T8 compared to T4. This supports the greater benefit of prolonged training. However, detraining decreased GLUT-4 expression, which is consistent with previous findings indicating that inactive skeletal muscle reduces GLUT-4 transcription and mRNA and protein levels [71]. Similar to IRS-1 and IGF-1, GLUT-4 expression improved by 74% with 8 weeks of PCA supplementation in AT4-PCA. This is inconsistent with in vitro findings that reported increased GLUT-4 expression following PCA treatment [24]. Additionally, PCA supplementation reduced the AUC of IPGTT in AT8-PCA (6215 ± 785) compared to AT8 (7020 ± 370) after 4 weeks of detraining at T12 (Supplementary Figure S2A,B). This indicates the potential counteracting effect of PCA on detraining. In brief, PCA improves glucose metabolism, regulates myokines, and mitigates the negative effects of detraining.

Besides the individual benefits of myokines and the insulin-signaling pathway in regulating glucose metabolism. It is crucial to identify the potential association between exercise-induced myokines and the insulin-signaling cascade to maintain glucose homeostasis. Therefore, the current study used grouping tools, including principal components analysis, cluster analysis, and discriminant function analysis, to investigate the underlying patterns and relationships among the studied variables. In principal component analysis, the second factor showed a high positive loading of IGF-1 and a high negative loading of myostatin. It is well established that myostatin and IGF-1 both show an important but opposite role in the regulation of growth and size of skeletal muscle and insulin-signaling pathways. IGF-1 promotes muscle hypertrophy and phosphorylation of PI3K and Akt while myostatin inhibits it. Likewise, an in vitro study demonstrated that myostatin decreased basal and insulin-induced IRS-1 tyrosine (Tyr495) phosphorylation, PI3K, Akt, and GLUT-4 mRNA and protein expression [72]. However, the addition of IGF-1 in muscle cells inhibits the negative effect induced by myostatin on the signaling pathway [73].

In addition, we identified high loading of PI3K, IL-6, and GLUT-4 in loading factors 4, 5, and 6 of the principal component analysis. Interestingly, previous evidence has also demonstrated a strong association between exercise-induced IL-6 expression with increased GLUT-4 translocation [74], PI3K-Akt activation [74], and glucose uptake [75]. These findings are well aligned with our results, further supporting the critical role of myokines in metabolic regulation.

Furthermore, the cluster analysis revealed three distinct clusters based on exercise durations and PCA supplementation. Cluster 1, representing 8 weeks of training, showed a higher mean value of IL-6 and a negative mean of myostatin. Enhanced IL-6 and reduced myostatin in response to 8 weeks of training are associated with improved GLUT-4 and P-Akt/Akt ratio, which are key metabolic markers of muscle metabolism and glucose uptake. Similarly, Ikeda et al. reported that increased IL-6 levels have a strong association with improved GLUT-4 expression. When animals were injected with an IL-6 neutralizing antibody before exercise, increased GLUT-4 expression and improved insulin sensitivity were not observed. These findings suggest that IL-6 is critical in the upregulation of GLUT-4 expression, and by neutralizing IL-6, the positive effect of exercise is canceled [76]. Our results also showed the importance of 8-week training in positively modulating IL-6 and myostatin secretions and their role in association with GLUT-4, thus improving glucose metabolism. Cluster 2 represents 4-week training and has shown elevated IGF-1 and IRS-2 levels, suggesting that even 4 weeks of exercise training is enough to stimulate IGF-1 and IRS-2. Cluster 3 is a representative of groups that received PCA supplementation. We noted that PCA supplementation has a positive role on irisin levels, FGF-21 levels, and IRS-1 expression, suggesting an altered metabolic response due to the PCA dose.

Moreover, discriminant function analysis identified IL-6, FGF-21, and IRS-1 as the most important predictors distinguishing between PCA and without PCA treatment groups. These predictors appear to play crucial roles in the metabolic responses, potentially highlighting the importance of PCA supplementation in regulating muscle secretions and glucose metabolism. The myokines IL-6 and FGF-21 are known for their metabolic roles in skeletal muscle metabolism, particularly by enhancing insulin sensitivity and glucose uptake. Our results are supported by previous studies that showed IL-6 and FGF-21 can interact with the insulin-signaling cascade by phosphorylating IRS-1 in skeletal muscle cells, thereby improving insulin action and increasing glucose uptake [77,78]. Additionally, our recent in vitro study showed that myokines improve glucose metabolism through communication between muscle, liver, and adipose tissue. PCA treatment further enhanced glucose metabolism and mitigated the negative effects of insulin resistance [79]. This indicates the potential role of PCA in improving glucose metabolism and highlights its potential as a therapeutic agent for metabolic health.

This study had some limitations. First, the sample size was relatively small, which may increase variability and reduce statistical significance. A larger sample size in future studies should yield more robust results. Second, the antioxidant capacity of PCA during exercise training and detraining was not assessed, considering that PCA possesses antioxidant properties. Additionally, exercise can activate the AMPK pathway, which may increase glucose uptake; however, the AMPK pathway was not examined in our study. Addressing these aspects in future studies could provide a more comprehensive understanding of the observed effects.

## 5. Conclusions

This study examined the impact of 4 weeks of moderate-intensity exercise training followed by 8 weeks of detraining and vice versa on myokines and insulin-signaling pathways. Notable improvements were observed in IGF-1, IL-6, FGF-21, myostatin, and insulin-signaling pathways (GLUT-4, PI3K, and IRS-2) after 4 weeks of training, but a greater improvement was seen after 8 weeks of training, including increased IGF-1 and IL-6 and decreased myostatin and FGF-21 levels. In addition to myokines, training increased PI3K, P-Akt/Akt, IRS-1, IRS-2, and GLUT-4 expression levels. In contrast, the beneficial effects of training were reduced after only 4 weeks of detraining. Therefore, it is better to continue physical activity training, as detraining could result in a reduction in the beneficial effects of training. PCA seemed to decrease the negative effect of detraining on myostatin and FGF-21 at T12 in AT8-PCA. In addition, the combined effects of PCA and training may offer greater benefits.

Our results offer new insights into the impact of training/detraining alone and in combination with PCA on the myokines and insulin-signaling pathways. These results suggest potential novel therapies, such as combined exercise and phenolic compound administration, for managing glucose metabolism and preventing or treating T2D. Further investigation is warranted to determine the optimal combination of training duration, polyphenol dose, and supplementation duration.

## Figures and Tables

**Table 1:** Eigenvalues of factors extracted with principal component analysis and varimax rotated factor loadings with significant variables in bold.

	Factor 1	Factor 2	Factor 3	Factor 4	Factor 5	Factor 6
Eigenvalue	3.177682	2.130712	1.648920	1.450948	1.166205	1.017794
% Total var.	21.18455	14.20475	10.99280	9.67298	7.77470	6.78529
Cumulative Eigenvalue	3.17768	5.30839	6.95731	8.40826	9.57447	10.59226
Cumulative %	21.18455	35.38929	46.38209	56.05508	63.82978	70.61507
Factor Loadings						
Training duration	0.467496	0.604667	0.061130	−0.093507	0.232490	0.368406
Detraining duration	0.563920	0.150715	0.586729	−0.009279	0.175967	0.162510
PCA	**0.968852**	0.001756	−0.058743	0.019085	0.037452	0.005106
FGF-21	−0.069646	−0.352479	0.216256	−0.161121	0.648786	−0.059321
IGF-1	−0.038731	**0.735340**	−0.068022	0.313606	−0.142595	0.022376
IL-6	0.204327	0.162538	−0.120818	0.054503	**0.710443**	0.225555
Myostatin	0.025372	**−0.869641**	0.031107	0.117826	−0.059805	0.020451
Irisin	−0.100065	−0.061525	−0.063804	−0.396537	−0.524290	0.027056
PI3K	−0.119980	0.139276	0.025471	**0.703937**	0.079711	0.155606
IRS-1	0.243344	−0.169831	0.070690	0.635818	−0.176945	−0.196094
IRS-2	−0.111179	0.359744	−0.670777	0.235372	−0.076692	−0.090543
GLUT-4	0.034674	0.036316	0.038942	0.008295	0.022137	**0.930953**
P-Akt	0.160373	0.254290	0.004893	−0.273040	0.498454	−0.288243
Group	0.450075	−0.041752	**−0.772618**	−0.221498	0.079374	0.047304
Expl. Var	2.788124	2.085171	1.479985	1.386836	1.611055	1.241089
Prp. Totl	0.185875	0.139011	0.098666	0.092456	0.107404	0.082739

**Table 2:** Cluster means.

	Cluster 1 *n* = 65	Cluster 2 *n* = 78	Cluster 3 *n* = 47
Exercise duration	0.883042	0.022045	−1.257814
Detraining duration	1.199994	−0.571681	−0.710818
PCA	0.797392	−0.414644	−0.414644
FGF-21	0.272150	−0.433520	0.343081
IGF-1	0.021389	0.481533	−0.828721
IL-6	0.465307	−0.151049	−0.392832
Myostatin	−0.069604	−0.649550	1.174237
Irisin	−0.299198	0.109226	0.232516
PI3K	0.002155	0.037973	−0.066000
IRS-1	0.133499	−0.217289	0.175981
IRS-2	−0.380456	0.5476708	−0.382736
GLUT-4	0.481973	−0.139146	−0.435635
P-Akt	0.280464	0.027416	−0.433375

**Table 3:** Discriminant function analysis summary.

	Wilks’ Lambda	Partial Lambda	F-Remove (1179)	*p*-Value	Toler.	1-Toler. (R-Sqr.)
FGF-21	0.890687	0.972180	5.12236	0.024820	0.747687	0.252313
IGF-1	0.872217	0.992767	1.30410	0.254991	0.612676	0.387324
IL-6	0.929458	0.931627	13.13707	0.000377	0.790123	0.209877
Myostatin	0.867070	0.998660	0.24027	0.624611	0.645224	0.354776
Irisin	0.866857	0.998906	0.19606	0.658457	0.933302	0.066698
PI3K	0.866631	0.999166	0.14935	0.699618	0.873500	0.126501
IRS-1	0.896774	0.965581	6.38067	0.012404	0.879700	0.120300
IRS-2	0.880657	0.983252	3.04899	0.082503	0.835026	0.164974
GLUT-4	0.866314	0.999531	0.08395	0.772355	0.941397	0.058603
P-Akt	0.874549	0.990120	1.78624	0.183081	0.915865	0.084135

## Data Availability

The original contributions presented in this study are included in the article and Supplementary Material. Further inquiries can be directed to the corresponding author.

## Supplementary Materials

The following supporting information can be downloaded at https://www.mdpi.com/article/10.3390/metabo15020087/s1, Figure S1: Blood lactate concentration. (**A**) Blood lactate concentration at a velocity of 15 m/min, 20 m/min, 25 m/min, and 30 m/min in the 4th week. (**B**) Blood lactate concentration in the AT8 group at a velocity of 15 m/min, 20 m/min, 25 m/min and 30 m/min at 8th week. (**C**) Blood lactate concentration in the AT8-PCA group at a velocity of 15 m/min, 20 m/min, 25 m/min, and 30 m/min in the 8th week. Data are shown as mean ± S.D. of duplicate experiments. ANOVA with multiple comparisons was performed, and statistical significance was set at *p* ≤ 0.05. Statistically significant difference among velocities * (*p* ≤ 0.05). Figure S2: Intraperitoneal glucose tolerance test (IPGTT). (**A**) An independent *t*-test was performed to analyze the difference between groups at T8. (**B**) An independent *t*-test was performed to analyze the difference between groups at T12. Data are shown as mean ± S.D. Statistical significance was set at *p* ≤ 0.05. Statistically significant * (*p* ≤ 0.05).
