# Supplementary material for: Separate and Combined Effects of Moderate-Intensity Exercise Training and Detraining with Protocatechuic Acid (PCA) on Myokines and Insulin-Signaling Pathways in Male Wistar Rats: A Preclinical Randomized Study
Source: Metabolites. 2025 Feb 1;15(2):87. doi: 10.3390/metabo15020087 (PMC11857820; doi:10.3390/metabo15020087)
Supplement: Supplementary file 1 [file metabolites-15-00087-s001.zip › metabolites-3378696-supplementary.pdf]

Supplementary Materials

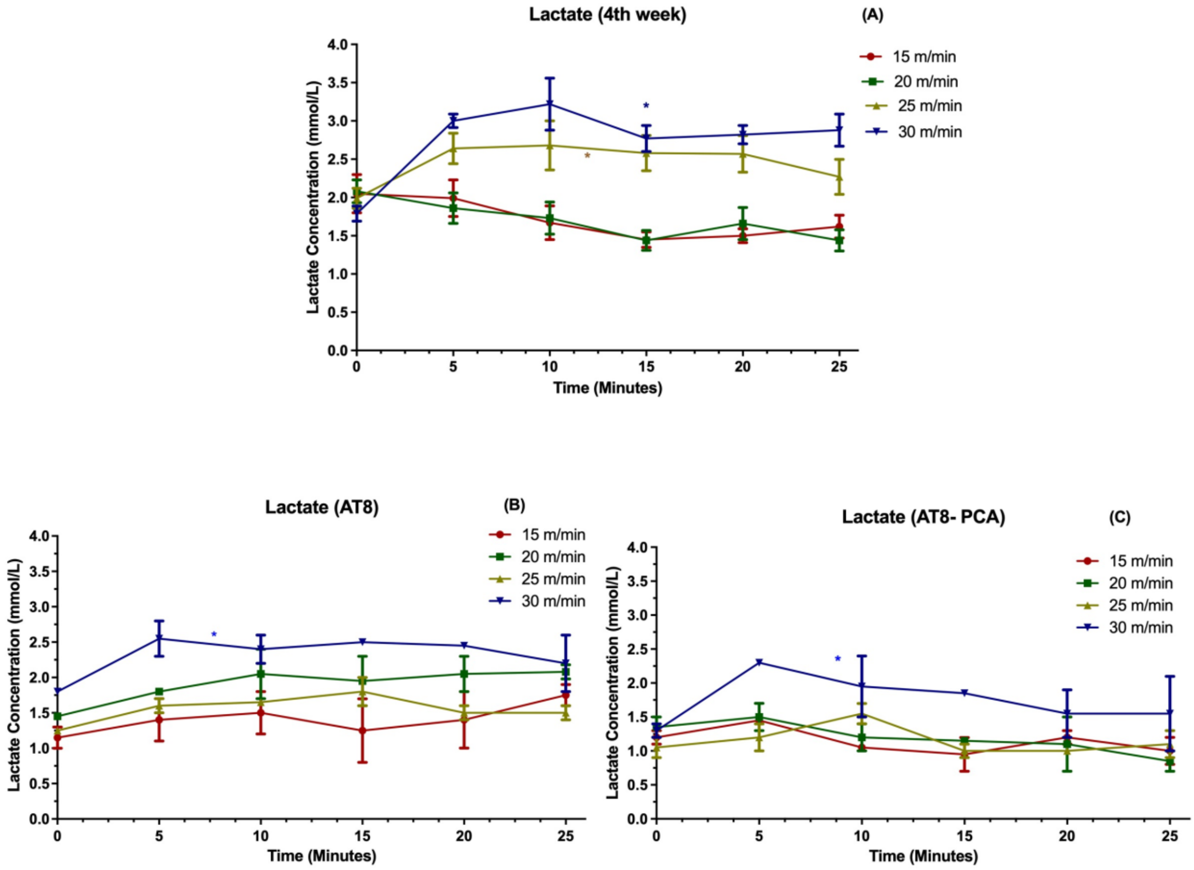

Figure S1. Blood lactate concentration. (**A**) Blood lactate concentration at a velocity of 15 m/min, 20 m/min, 25 m/min and 30 m/min at 4th week, (**B**) Blood lactate concentration in AT8 group at a velocity of 15 m/min, 20 m/min, 25 m/min and 30 m/min at 8th week, (**C**) Blood lactate concentration in AT8-PCA group at a velocity of 15 m/min, 20 m/min, 25 m/min and 30 m/min at 8th week. Data are shown as Mean ± S.D. of duplicate experiments. ANOVA with multiple comparisons was performed, and statistical significance was set at $p \leq 0.05$. Statistically significant difference among velocities * ($p \leq 0.05$).

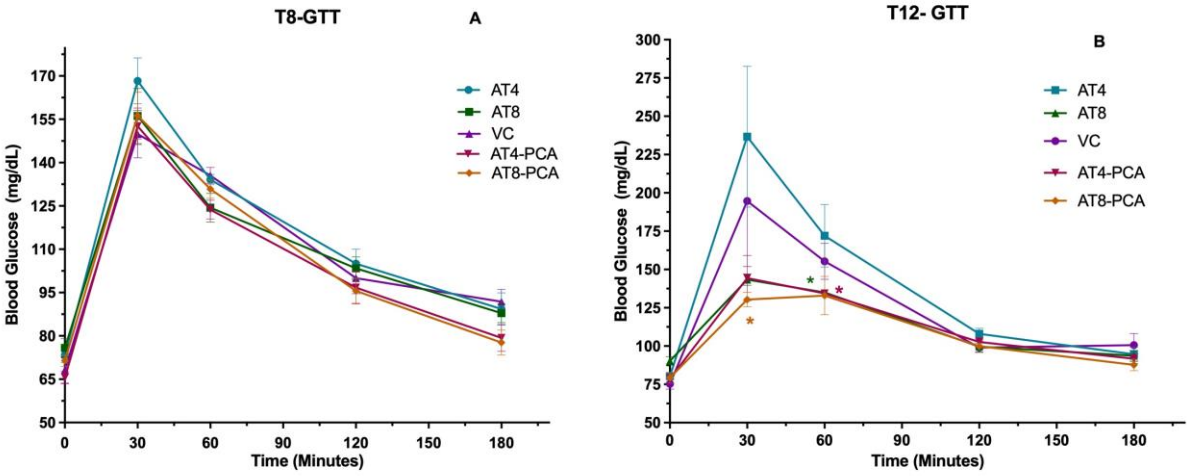

Figure S2. Intraperitoneal glucose tolerance test (IPGTT). (**A**) An independent t-test was performed to analyze the difference between groups at T8. (**B**) An independent t-test was performed to analyze the difference between groups at T12. Data are shown as Mean ± S.D. Statistical significance was set at $p \leq 0.05$. Statistically significant * ($p \leq 0.05$).
